# Meningeal immunity and “Interstitial” therapy: a new paradigm for immunotherapy in glioblastoma

**DOI:** 10.3389/fimmu.2026.1847377

**Published:** 2026-06-15

**Authors:** Hongan Fei, Xichao Wen, Wensong Wu, HaiPeng Liu, Haipeng Xie, Yan Wang, Kebin Zheng, Zhaomu Zeng, Zetong Bai

**Affiliations:** 1 Department of Neurosurgery, Affiliated Hospital of Hebei University, Baoding, China; 2 Department of Neurosurgery, Jiangxi Provincial People’s Hospital, The First Affiliated Hospital of Nanchang Medical College, Nanchang, China

**Keywords:** blood-brain barrier, glioblastoma, immunotherapy, interstitial therapy, meningeal immunity, tissue-resident macrophages

## Abstract

Glioblastoma (GBM) is one of the most aggressive brain tumors, and its immunotherapy faces significant challenges, including the physical barrier of the blood-brain barrier, the tumor immunosuppressive microenvironment, and the difficulty of effective immune cell infiltration into tumor tissue. In recent years, the anatomical and functional basis of the meningeal immune system has been gradually revealed, particularly the discovery of the dural lymphatic system and tissue-resident border-associated macrophages (rBAMs), providing new insights for GBM immunotherapy. “Interstitial” immunotherapy, as an innovative strategy, emphasizes a shift from the traditional focus on the tumor interior to the tumor “border,” leveraging the unique advantages of the meningeal immune system to enhance the effectiveness of immune responses. This review systematically examines the current status and dilemmas of GBM immunotherapy, the anatomical and functional basis of meningeal immunity, the discovery and mechanisms of rBAMs, the theoretical implications of “interstitial” immunotherapy, and the clinical translation prospects and challenges of this strategy, aiming to provide new theoretical support and practical guidance for GBM immunotherapy.

## 1 Introduction

Glioblastoma represents the most common and aggressive primary malignant brain tumor in adults, characterized by rapid growth, diffuse infiltration, and a notoriously poor prognosis despite multimodal standard treatments including maximal surgical resection, radiotherapy, and chemotherapy (1). The median survival remains dismal at approximately 14–15 months, with only about 26.5% of patients surviving beyond two years (1, 2). The clinical challenge posed by GBM arises from its complex biology, including pronounced intratumoral heterogeneity, adaptive resistance mechanisms, and an immunosuppressive tumor microenvironment (TME) that collectively hinder therapeutic efficacy. Traditional modalities have shown limited success in significantly improving patient outcomes, underscoring the urgent need for novel therapeutic strategies (3).

Immunotherapy, which harnesses the host immune system to recognize and eradicate tumor cells, has revolutionized treatment paradigms in several solid and hematologic malignancies such as melanoma and non-small cell lung cancer (2, 4). This success has spurred considerable interest in applying immunotherapeutic approaches to GBM. Various modalities including immune checkpoint inhibitors (ICIs), therapeutic vaccines, adoptive cell therapies such as chimeric antigen receptor (CAR) T cells, and oncolytic virotherapy have been explored in preclinical and clinical settings (5–7). However, the efficacy of these immunotherapies in GBM has been largely disappointing, with many trials failing to demonstrate significant survival benefits (5) (3). The unique immunological landscape of the central nervous system (CNS), historically considered an immune-privileged site, contributes to this challenge (1).

Several biological barriers impede effective immunotherapy in GBM. The blood-brain barrier (BBB) restricts the infiltration of immune effector cells and the delivery of therapeutic agents into the brain parenchyma (8). Furthermore, the GBM TME is characterized by profound immunosuppression mediated by tumor-associated macrophages, myeloid-derived suppressor cells, and regulatory T cells, which collectively dampen anti-tumor immune responses (9). Metabolic reprogramming within the tumor further exacerbates immunosuppression by altering nutrient availability and immune cell function (10). Additionally, GBM tumors employ immune escape mechanisms including heterogeneity, and adaptive resistance to evade immune surveillance and immunotherapeutic interventions (1, 11).

Recent advances in understanding the immune landscape of the CNS have challenged the notion of strict immune privilege. The discovery of meningeal lymphatic vessels and the characterization of border-associated macrophages (BAMs) have revealed that the meninges serve as critical immunological interfaces connecting the CNS with the peripheral immune system (12, 13). These meningeal immune niches harbor diverse populations of resident and infiltrating immune cells, including tissue-resident macrophages, dendritic cells, and T lymphocytes, which participate in immune surveillance and neuroimmune communication (14). The meninges thus represent a dynamic immune environment capable of modulating CNS immunity and potentially influencing tumor-immune interactions in GBM (15).

Among the meningeal immune cells, resident border-associated macrophages have emerged as key players in CNS immune regulation. These specialized macrophages reside in the meninges, perivascular spaces, and choroid plexus, where they contribute to tissue homeostasis, immune surveillance, and response to pathology (16, 17). Recent studies have highlighted the role of rBAMs in modulating neuroinflammation and interacting with infiltrating immune cells, shaping the immunological milieu at the CNS borders (18). Importantly, these cells possess antigen-presenting capacity and can influence T cell activation and trafficking, suggesting their potential involvement in anti-tumor immunity within the CNS (15). The identification of rBAM subsets with distinct molecular signatures and functional properties provides new insight into the complexity of meningeal immunity and its impact on CNS diseases including GBM.

The recognition of the meninges as an immunologically active compartment has opened novel avenues for therapeutic intervention in GBM. Meningeal vascular blockage can expand the rBAM pool, promote T cell activation at the dural interface, and enhance intratumoral cytotoxic T cell responses against glioblastoma (15). Strategies such as meningeal blood vessel blockage have been shown to expand rBAMs and potentiate T cell-mediated anti-glioblastoma immunity in preclinical models, and rBAM abundance is correlated with improved survival in glioblastoma patients (15). These findings suggest that the meningeal immune system serves as a critical gatekeeper and amplifier of CNS anti-tumor immunity, offering a promising target to overcome the immunosuppressive barriers characteristic of GBM (15).

Building on this understanding, meningeal vascular blockage has been proposed as a surgical strategy to enhance immunotherapeutic efficacy in GBM by modulating the immune microenvironment at the CNS borders, particularly the meninges (15). This approach aims to leverage the unique immunological properties of the meningeal compartment, including the antigen-presenting capacity of rBAMs and their interactions with T cells, to break the tumor-induced immunosuppressive shield and facilitate robust anti-tumor immune responses (15). By targeting the meningeal immune niche, interstitial immunotherapy may overcome limitations imposed by the BBB and the immunosuppressive TME, representing a novel paradigm in GBM immunotherapy.

In summary, the dismal prognosis of GBM underscores the urgent need for innovative therapeutic strategies beyond conventional modalities. Immunotherapy holds promise but faces formidable challenges due to the unique CNS immune environment and GBM-associated immunosuppression. The meninges, as a critical interface between the CNS and peripheral immunity, harbor specialized immune populations such as rBAMs that regulate immune surveillance and response. Harnessing the meningeal immune system through interstitial immunotherapy offers a new frontier to enhance anti-glioblastoma immunity and improve patient outcomes. This review systematically examines the challenges of GBM immunotherapy, the anatomy and function of meningeal immunity, the mechanistic roles of rBAMs, and the emerging concept of interstitial immunotherapy with its translational potential.

## 2 The dilemma of GBM immunotherapy

### 2.1 Physical and physiological barriers of the blood-brain barrier

The blood-brain barrier is a highly specialized and complex structure that serves as a critical physical and physiological barrier between the circulating blood and the central nervous system. It is primarily composed of brain microvascular endothelial cells connected by tight junctions, supported by the basal lamina, astrocytic endfeet, and pericytes, which collectively form the neurovascular unit. This architecture protects neural tissue from toxins and pathogens (19). The endothelial tight junction proteins such as claudins, occludin, and junctional adhesion molecules are key elements that maintain the selective permeability of the BBB. The astrocytes and pericytes further regulate the barrier’s integrity and function, influencing both paracellular and transcellular transport mechanisms.

In the context of glioblastoma, the BBB undergoes heterogeneous disruption, particularly in the tumor core where the blood-brain-tumor barrier (BBTB) forms. Although the BBTB exhibits increased permeability compared to the intact BBB, it remains a significant obstacle to the infiltration of immune effector cells such as CAR-T cells and T lymphocytes, as well as to the delivery of therapeutic agents. Studies have demonstrated that despite partial BBB breakdown near GBM regions, the barrier function is largely preserved in the peritumoral areas, limiting the access of systemically administered immunotherapies and chemotherapeutics to infiltrative tumor cells that reside beyond the core lesion. This spatial heterogeneity in BBB integrity contributes to the therapeutic resistance and recurrence characteristic of GBM.

The restrictive nature of the BBB significantly impairs the efficacy of systemic immunotherapies and chemotherapeutic drugs by limiting their penetration into the brain tumor microenvironment. Many therapeutic molecules, including large biologics, cannot effectively traverse the BBB in sufficient concentrations to exert their anti-tumor effects (20, 21). This has prompted extensive research into novel strategies to enhance BBB permeability or to develop delivery systems capable of crossing the BBB. Approaches such as focused ultrasound have shown promise in transiently disrupting the BBB to facilitate drug delivery (22, 23). Similarly, nanoparticle-based delivery systems functionalized with targeting ligands (e.g., Angiopep-2, LDLR ligands) have been engineered to exploit receptor-mediated transcytosis pathways to improve BBB penetration and tumor targeting (24, 25).

Furthermore, the BBB’s barrier function is modulated by molecular signaling within the tumor microenvironment. For instance, glioblastoma cells secrete factors such as interleukin-6 (IL-6) that activate STAT3 signaling in BBB endothelial cells, leading to downregulation of tight junction proteins and increased permeability (26). However, this increased permeability is localized and does not fully alleviate the delivery challenges posed by the BBB. Additionally, pericytes, which are critical for BBB integrity, undergo phenotypic changes induced by tumor-secreted transforming growth factor-beta (TGF-β), contributing to barrier dysfunction and aberrant vasculature in GBM (27, 28). These pathophysiological alterations further complicate the delivery of therapeutics and immune cells across the BBB.

Given these complexities, the BBB remains a formidable physical and physiological barrier in GBM therapy. The development of *in vitro* BBB models incorporating endothelial cells, pericytes, and astrocytes has provided valuable platforms for studying BBB dynamics and screening drug delivery strategies (29, 30). Such models have highlighted the importance of maintaining tight junction integrity and physiological shear stress to replicate *in vivo* BBB properties accurately. Combining these insights with advanced drug delivery technologies and barrier modulation techniques holds promise for overcoming the BBB’s impediments to effective GBM immunotherapy and chemotherapy.

In summary, the BBB’s intricate structure and selective permeability form a critical barrier that restricts immune cell infiltration and drug delivery to GBM lesions. Despite partial disruption in tumor regions, the BBB continues to limit therapeutic efficacy, necessitating innovative approaches to traverse or modulate this barrier. Strategies integrating nanotechnology, focused ultrasound, receptor-mediated transport, and molecular modulation of BBB components are being actively explored to enhance drug and immune effector access to the brain tumor microenvironment, potentially improving clinical outcomes for GBM patients.

### 2.2 Complexity of the tumor immunosuppressive microenvironment

The glioblastoma tumor microenvironment is notoriously complex and heavily immunosuppressive, posing a significant barrier to effective immunotherapy. A hallmark of this microenvironment is the abundant infiltration of immunosuppressive cells, principally tumor-associated macrophages (TAMs), regulatory T cells (Tregs), and myeloid-derived suppressor cells (MDSCs). These cells not only constitute a major portion of the non-neoplastic cellular population within GBM but actively contribute to immune evasion and tumor progression. For instance, TAMs participate in immune suppression and tumor progression in GBM (31–33). The immunosuppressive nature of TAMs is further underscored in GBM (34, 35). The presence of Tregs, characterized by FOXP3 expression, is markedly increased in GBM tissues, where they exert immunosuppressive effects in the tumor microenvironment (36, 37). Moreover, MDSCs accumulate in tumor sites of GBM patients, exerting potent immunosuppressive effects by inhibiting T cell proliferation and function through adenosine and iNOS pathways (38). The coordinated action of these immunosuppressive cells establishes a formidable barrier to anti-tumor immunity, contributing to the resistance of GBM to conventional therapies and immune checkpoint inhibitors.

In addition to cellular components, TGF-β signaling in the GBM microenvironment can drive tumor progression and inhibit T cell activity (39). In GBM, PAK4 in tumor cells mediates TGF-β1 release, which drives CD8+ T cell dysfunction and impairs anti-tumor immunity (40). IL-10 contributes to the suppression of antigen presentation and the skewing of macrophages toward an immunosuppressive phenotype. This cytokine milieu synergizes with immune checkpoint molecules such as PD-L1, which is frequently overexpressed on infiltrating immune cells in GBM (41). LAG3 is a key driver of T cell exhaustion in glioblastoma (42). The multifaceted nature of this immunosuppressive network underscores the challenge of reversing immune dysfunction in GBM.

Recent studies have highlighted the heterogeneity and plasticity of TAMs and MDSCs within the GBM microenvironment, revealing distinct subpopulations with varying immunosuppressive capacities and metabolic profiles (43, 44). For example, monocytic MDSCs (mMDSCs) expressing CCR2 and CX3CR1 have been shown to infiltrate the tumor and suppress CD8+ T cell function via nitric oxide synthase-dependent mechanisms (38, 45). Targeting these myeloid populations through inhibition of key pathways such as CSF1R signaling or metabolic enzymes like ACAT1 has been proposed as a potential strategy to reprogram the TME and enhance anti-tumor immunity in GBM. Furthermore, the recruitment and polarization of TAMs are influenced by tumor-derived factors such as MDK and EGFRvIII-mediated signaling, which promote M2-like phenotypes and secretion of immune-suppressive chemokines like CXCL1, facilitating immune escape and tumor progression (46). These findings suggest that myeloid cells are not only passive components but active drivers of the immunosuppressive microenvironment in GBM.

The interplay between tumor cells, immunosuppressive cells, and soluble mediators creates a dynamic and resilient network that supports tumor immune evasion. Notably, glioma stem-like cells (GSCs) contribute to this immunosuppressive milieu by secreting factors such as OLFML3 and IL-19, which recruit and modulate microglia and macrophages, enhancing their suppressive functions (47, 48). Additionally, tryptophan metabolism via IDO1 and the kynurenine pathway within the TME contributes to T cell exhaustion, further promoting immune tolerance (49, 50). Epigenetic regulators such as KDM6B in myeloid cells also play a role in maintaining immunosuppressive phenotypes, and their inhibition has been shown to sensitize GBM to immune checkpoint blockade (51). Collectively, these diverse mechanisms highlight the complexity of the GBM immunosuppressive microenvironment and underscore the need for multifaceted therapeutic strategies targeting both cellular and molecular components.

In summary, the GBM tumor microenvironment is characterized by a highly immunosuppressive network composed of TAMs, Tregs, MDSCs, immunosuppressive cytokines, and immune checkpoint molecules that collectively inhibit effective anti-tumor immunity. The heterogeneity and plasticity of these components, along with tumor-driven metabolic and epigenetic reprogramming, contribute to the profound immune evasion observed in GBM. Understanding these intricate interactions provides a critical foundation for developing novel immunotherapeutic approaches aimed at remodeling the tumor microenvironment to restore immune surveillance and improve patient outcomes (9, 52–54).

### 2.3 CAR-T cells and challenges of T cell infiltration

Chimeric antigen receptor T cell therapy has emerged as a revolutionary immunotherapeutic approach with remarkable success in hematological malignancies, yet its application in glioblastoma faces significant hurdles. One of the primary challenges in GBM treatment with CAR-T cells is the pronounced tumor antigen heterogeneity and immune escape mechanisms inherent to the tumor. GBM exhibits diverse and dynamic expression of tumor-associated antigens (TAAs), such as EGFRvIII, IL13Rα2, HER2, and others, which complicates the targeting by CAR-T cells designed against a single antigen. This heterogeneity allows tumor cells lacking the targeted antigen to evade immune surveillance, leading to treatment resistance and tumor relapse (55). Moreover, the immunosuppressive tumor microenvironment in GBM is a key challenge for CAR-T cell therapy efficacy (56).

Another formidable barrier to CAR-T cell efficacy in GBM is the restricted trafficking and infiltration of immune cells into the tumor site. The blood-brain barrier and blood-tumor barrier (BTB) are physiological obstacles that limit the passage of circulating immune cells, including CAR-T cells, into the central nervous system tumor parenchyma. Even when CAR-T cells are systemically administered, their ability to cross the BBB and accumulate in sufficient numbers within the tumor is limited, resulting in suboptimal antitumor activity. Clinical studies have shown that intracerebral or locoregional administration of CAR-T cells has demonstrated safety and feasibility with preliminary evidence of response in glioblastoma patients (57, 58). Furthermore, the immunosuppressive milieu within the tumor, characterized by altered tumor metabolism, restricts CAR-T cell motility, limiting their antitumor response (59).

The persistence and functional longevity of CAR-T cells within the GBM TME also constitute a critical limitation. Immune cells infiltrating GBM often exhibit an exhausted phenotype, with diminished cytotoxicity and proliferative capacity. CAR-T cells in GBM frequently demonstrate reduced survival and impaired effector functions due to chronic antigen exposure and the suppressive TME. Strategies to enhance CAR-T cell persistence include genetic modifications to resist exhaustion, co-expression of cytokines to support T cell survival, and combination therapies with immune checkpoint inhibitors to restore T cell functionality (60, 61). Additionally, the short *in vivo* lifespan of CAR-T cells in GBM contrasts with their prolonged persistence in hematological malignancies, underscoring the need for innovative approaches to sustain their activity in the CNS environment.

In summary, CAR-T cell therapy in glioblastoma is challenged by tumor antigen heterogeneity, limited trafficking across the BBB, and the immunosuppressive microenvironment that restricts T cell infiltration and persistence. Addressing these barriers through multi-antigen targeting, optimized delivery methods, and engineering CAR-T cells to resist exhaustion and immunosuppression is essential to improve therapeutic efficacy. Emerging evidence also suggests that targeting meningeal lymphatic function may enhance anti-tumor immunity, providing an additional avenue to overcome these challenges (62). These multifaceted obstacles highlight the complexity of implementing CAR-T cell therapy in GBM and the necessity for integrated strategies to realize its full potential.

### 2.4 Clinical application limitations of immunotherapy

Immunotherapy has emerged as a promising modality for treating various malignancies, including glioblastoma, the most common and aggressive primary brain tumor in adults. Despite significant advances in immunotherapeutic strategies such as immune checkpoint inhibitors, vaccines, adoptive cell therapies, and oncolytic virotherapy, their clinical efficacy in GBM remains limited. One major factor contributing to this limited success is the unique and highly immunosuppressive tumor immune microenvironment (TIME) characteristic of GBM. The GBM immune microenvironment is shaped by brain immune privilege and tumor-intrinsic immune escape mechanisms to create a tumor-promoting microenvironment (1). The GBM TIME is dominated by immunosuppressive cell populations including regulatory T cells, tumor-associated macrophages, and myeloid-derived suppressor cells, which collectively impair the activation and infiltration of effector T cells. Additionally, GBM tumors express immune checkpoint molecules such as PD-L1 and CTLA-4, further dampening anti-tumor immunity. These factors create a formidable barrier to effective immunotherapy, resulting in poor clinical responses despite promising preclinical data.

Another critical limitation in the clinical application of immunotherapy for GBM is the risk of immune-related adverse events (irAEs), particularly neuroinflammation. The brain’s delicate environment is highly susceptible to inflammation-induced damage, and immune activation within the CNS can provoke cerebral edema, seizures, and other neurological toxicities. These adverse effects constrain the dosing and scheduling of immunotherapeutic agents, limiting their therapeutic window. For example, clinical trials involving ICIs such as nivolumab and pembrolizumab have reported manageable immune-related adverse events (63, 64). The risk of irAEs is further amplified in combination therapies, which, although potentially more efficacious, increase the complexity of immune modulation and toxicity management. Therefore, the balance between anti-tumor efficacy and safety remains a significant challenge in GBM immunotherapy clinical design.

**Figure 1:**
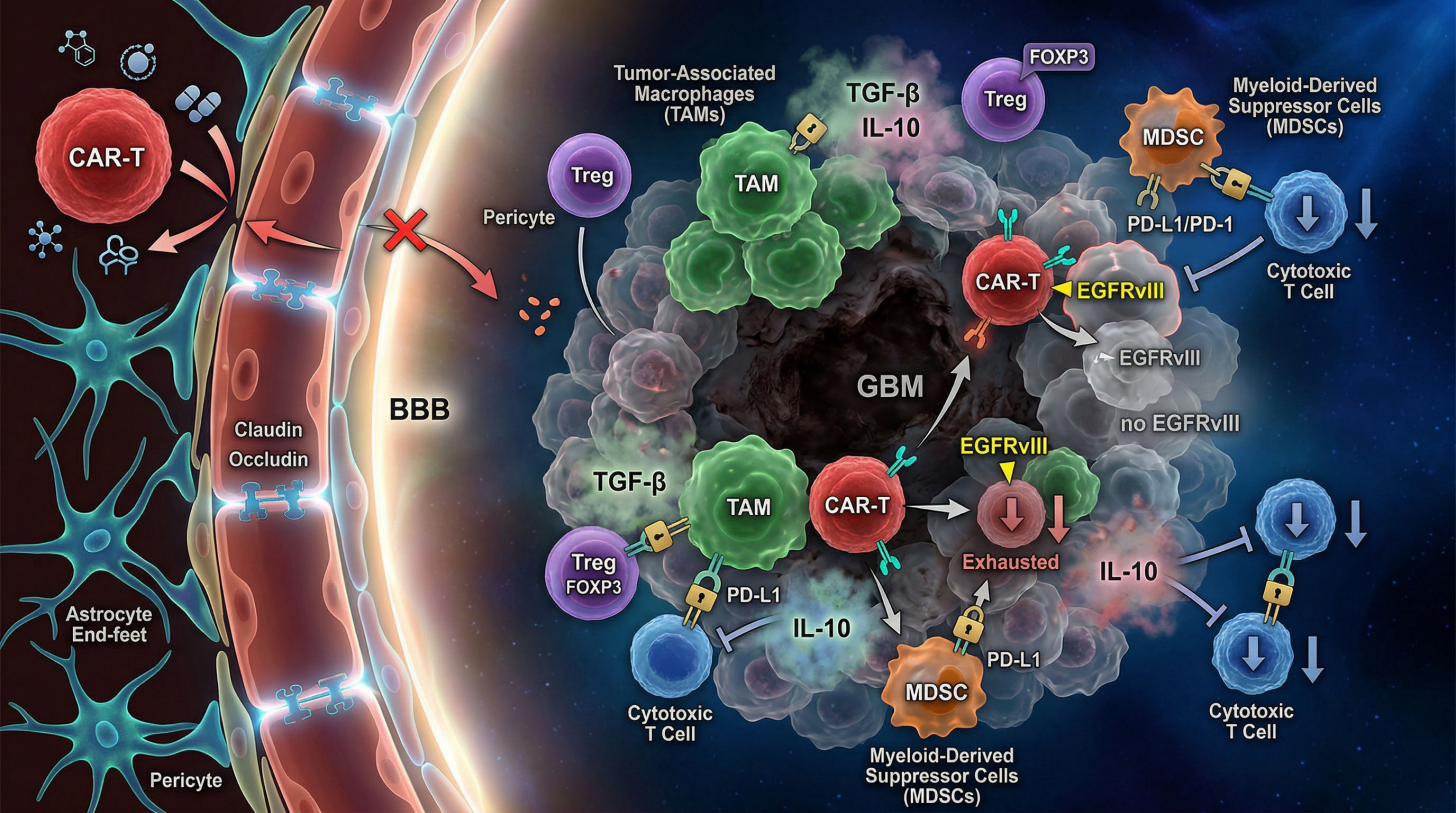
Multifaceted barriers to glioblastoma immunotherapy.

Moreover, the lack of effective targeting strategies and delivery systems for immune cells and immunotherapeutic agents significantly hampers clinical translation. The BBB restricts the penetration of large molecules such as monoclonal antibodies and cellular therapies into the tumor site, reducing their bioavailability and efficacy. While CAR-T cell immunotherapies have yielded some results in preclinical and clinical studies for GBM, and convection-enhanced delivery has been proposed as an optimization strategy for CAR-T therapy, the clinical utility of these approaches and nanomedicine-based carriers is still under evaluation (65). Additionally, the immunosuppressive microenvironment within GBM often leads to the functional exhaustion of infiltrating immune cells, limiting the persistence and cytotoxicity of adoptively transferred cells (53, 66). The heterogeneity of GBM antigens and the dynamic evolution of tumor clones further complicate the design of targeted immunotherapies. For instance, tumor antigen heterogeneity challenges the efficacy of CAR T-cell therapies (67). The development of bispecific antibodies aims to address these issues, although delivery efficiency and immune-related adverse events remain limitations in GBM treatment (68). Therefore, the absence of robust delivery platforms and precise targeting modalities remains a bottleneck in translating immunotherapeutic advances into effective GBM treatments (**Figure 1**).

In summary, the clinical application of immunotherapy in glioblastoma faces multifaceted limitations. The immunosuppressive tumor microenvironment, characterized by abundant suppressive immune cells and checkpoint molecule expression, undermines therapeutic efficacy. The risk of immune-related adverse events, especially neuroinflammation, restricts treatment intensity and complicates regimen design. Furthermore, challenges in delivering immunotherapeutic agents across the blood-brain barrier and achieving sustained immune cell function within the tumor impede clinical success. Future progress depends on integrating multimodal strategies that modulate the tumor microenvironment, enhance immune cell infiltration and function, and employ innovative delivery systems to overcome these hurdles. Personalized approaches guided by biomarkers may help optimize patient selection for immunotherapy in GBM (4, 6, 69).

## 3 Anatomical and functional basis of meningeal immunity

### 3.1 Structure and function of the dural lymphatic system

The dural lymphatic system is a recently characterized network of lymphatic vessels located within the dura mater, the outermost meningeal layer enveloping the brain. This system serves as a critical conduit for the drainage of cerebrospinal fluid (CSF) and immune cells, thereby connecting the central nervous system immune environment with the peripheral lymphatic system. Anatomically, meningeal lymphatic vessels (MLVs) are predominantly situated along the dural venous sinuses, such as the superior sagittal sinus and transverse sinuses, where they form an intricate network facilitating fluid and cellular exchange (70, 71). Advanced imaging studies using 7T MRI have delineated the morphology of these vessels in humans, revealing their concentration along the superior sagittal sinus and cortical veins, with largely fixed enhancement patterns suggestive of potential transport of proteinaceous fluid (71). Furthermore, immunohistochemical investigations have demonstrated widespread distribution of lymphatic vessels in human dura mater, which can be found adjacent to and distant from blood vessels, underscoring the extensive anatomical reach of the dural lymphatic system (72, 73).

Functionally, the dural lymphatic vessels act as a drainage pathway for CSF and interstitial fluid (ISF), facilitating the removal of metabolic waste and antigens from the brain parenchyma to deep cervical lymph nodes (dCLNs). This drainage supports immune surveillance by transporting antigen-presenting cells from the CNS to peripheral lymphoid tissues, where immune activation is modulated (70). The dural lymphatics thereby maintain CNS homeostasis by coupling fluid clearance with immune cell trafficking. The glymphatic system, a complementary perivascular pathway involving astrocytic aquaporin-4 water channels, works in concert with the dural lymphatics to distribute CSF and clear metabolic byproducts, highlighting a coordinated neurofluid clearance mechanism (74, 75). Notably, the presence of lymphatic endothelium in dural spaces near arachnoid granulations supports the role of dura-associated structures in CSF resorption, and arachnoid cuff exit points are newly identified meningeal structures that contribute to neuroimmune interactions (76–78).

Interestingly, recent studies have revealed that the dural lymphatic vessels exhibit anatomical and molecular features characteristic of initial lymphatic vessels, such as the absence of smooth muscle and limited valve structures, which may allow bidirectional fluid movement under certain conditions (79, 80). This unique physiology could have implications for nanoparticle transport and targeted drug delivery to the brain, as nanoparticles administered to deep cervical lymph nodes were shown to reach the meninges and brain through lymphatic pathways (81). Additionally, fibroblasts within the dura mater suggest a complex immunological niche that supports immune cell recruitment, retention, and activation, further underscoring the immunocompetent nature of the dural environment (82, 83).

Collectively, the dural lymphatic system serves as a pivotal anatomical and functional interface between the CNS and peripheral immune system, mediating neurofluid clearance, immune surveillance, and neuroimmune interactions. Its dysfunction is increasingly recognized as a contributor to CNS diseases and tumor immune evasion. Future research focusing on the modulation of dural lymphatic function may open new avenues for therapeutic interventions in neurodegenerative diseases, brain tumors, and neuroinflammatory disorders. The extensive anatomical distribution and dynamic immune roles of the dural lymphatics position them as a promising target for advancing CNS disease treatment strategies.

### 3.2 Composition and distribution of meningeal immune cells

The meninges, composed of the dura mater, arachnoid mater, and pia mater, harbor a diverse and specialized immune cell population that forms a unique immunological microenvironment essential for central nervous system surveillance and homeostasis. Various studies have characterized the meningeal immune landscape, revealing that it contains an extensive repertoire of immune cells that participate in immune surveillance, post-injury recovery, and responses to chronic neurodegenerative diseases, maintaining CNS integrity and responding to pathological challenges (84, 85).

Macrophages constitute a significant portion of meningeal immune cells and are distributed across different meningeal layers, including the dura and leptomeninges. These macrophages are heterogeneous, comprising border-associated macrophages such as dural, leptomeningeal, perivascular, and choroid plexus macrophages, each with distinct origins, phenotypes, and functions (86, 87). For instance, leptomeningeal macrophages strongly express CD163 (88). Notably, in ischemic stroke, meningeal macrophages infiltrate Virchow-Robin perivascular spaces and interact with PDGFR-β-positive adventitial fibroblasts, and in bacterial meningitis induced by Escherichia coli, meningeal macrophages undergo characteristic transcriptomic changes in response to neuroinflammation (89, 90). The dura mater also contains a distinct subset of macrophages, including extrasinusoidal dural macrophages, which differ transcriptionally and functionally from leptomeningeal macrophages and are implicated in efferocytosis during autoimmune neuroinflammation (91).

Dendritic cells and mast cells further enrich the meningeal immune milieu. Mast cells, residing predominantly in the dura, serve as tissue-resident sentinels that respond robustly to viral infections by releasing cytokines and chemokines, thereby enhancing antiviral immunity (92). The strategic localization of mast cells near meningeal vasculature enables them to regulate cerebrospinal fluid dynamics and mediate immune cell recruitment, highlighting their pivotal role at the brain-dura interface (93).

Adaptive immune cells, particularly T and B lymphocytes, are resident within the meninges and contribute to CNS immune surveillance and regulation. In human leptomeninges, most T cells are CD8+ and exhibit tissue-resident memory T cell characteristics (17, 94). B cells within the meninges are involved in humoral immunity, with evidence suggesting their participation in regulating meningeal myeloid cell activation and influencing behavior under stress conditions (95). Moreover, meningeal B cell populations can expand and produce immunoglobulins, which have been implicated in Rett syndrome, indicating their importance in neuroimmune interactions (96).

Spatially, meningeal immune cells are often concentrated in border regions such as around dural venous sinuses and cranial nerves, forming specialized immune hubs (80). These immune cells reside predominantly in the meninges rather than the brain parenchyma, enabling them to act as first responders to CNS insults without compromising the immune privilege of the brain tissue itself (97). The arachnoid barrier acts as a boundary between the CNS and dura, containing ACE points that serve as trans-barrier pathways allowing exchange of substances and immune cells between compartments (98). Interestingly, anatomical structures such as arachnoid cuff exit (ACE) points provide discontinuities in the arachnoid barrier that allow selective exchange of fluids and immune cells between the dura and subarachnoid space, serving as critical routes for neuroimmune communication (98, 99).

The meningeal immune compartment is not static; it dynamically adapts to physiological and pathological conditions. During neuroinflammation, immune cell composition and activation states shift markedly. For example, bacterial meningitis triggers infiltration and activation of neutrophils, macrophages, dendritic cells, and T cells in the meninges, with specific immune signatures evolving across infection stages (100, 100). Similarly, in neurodegenerative diseases such as multiple sclerosis (MS), complement activation in the leptomeninges and accumulation of C5a receptor-positive microglia/macrophages contribute to compartmentalized inflammation and cortical pathology (101). Aging also influences meningeal immunity, and single-cell profiling has revealed changes in leptomeningeal cell profiles in aged individuals associated with Alzheimer’s disease (17).

Taken together, the meningeal immune system comprises a complex and heterogeneous assembly of innate and adaptive immune cells strategically localized in meningeal layers, particularly at border regions. These cells provide critical surveillance, mediate immune responses to CNS insults, and maintain CNS immune homeostasis. The unique localization and specialized functions of meningeal immune cells position them as key players in CNS health and disease, with emerging evidence supporting their potential as therapeutic targets in neuroinflammatory and neurodegenerative disorders (77, 84). This intricate meningeal immune microenvironment underscores the meninges as an active immunological interface rather than a mere protective barrier.

### 3.3 Immune communication mechanisms between the meninges and brain parenchyma

The meninges and brain parenchyma engage in a dynamic immune communication network facilitated by cytokines, chemokines, and lymphatic pathways that collectively maintain central nervous system homeostasis and coordinate immune surveillance. Although the CNS parenchyma itself contains relatively few lymphocytes and exhibits limited antigen-presenting capacity at steady state, the surrounding meninges host diverse immune cell populations that actively participate in immune responses (102). This spatial separation allows the meninges to serve as an immunological interface, where soluble mediators such as cytokines and chemokines are secreted to modulate the activity of resident and infiltrating immune cells both within the meninges and the brain parenchyma. For instance, LPS-induced systemic inflammation leads to elevated expression of pro-inflammatory cytokines including interleukin-1β (IL-1β) in myeloid cells at meningeal and other CNS borders, indicating the meningeal compartment’s role in shaping CNS immune responses (103).

Central to this communication is the meningeal lymphatic system (MLS), a network of lymphatic vessels that drains cerebrospinal fluid from the CNS to the deep cervical lymph nodes (70). The MLS thus establishes a physical conduit for immune surveillance, enabling CNS-derived antigens and immune cells to reach peripheral lymphoid tissues, where adaptive immune responses can be initiated or modulated. The glymphatic system complements this by facilitating perivascular exchange of CSF and interstitial fluid within the brain parenchyma, primarily mediated by astrocytic aquaporin-4 channels, thereby supporting solute clearance and immune signaling (104) (105). The integration of these systems suggests a coordinated pathway by which immune signals and cells transit between the brain and peripheral immune compartments, challenging the traditional notion of the brain as an immune-privileged organ.

Importantly, immune cells can migrate from the meninges into the brain parenchyma via the meningeal lymphatic vessels and associated pathways. Meningeal lymphatics not only drain immune cells but also actively recruit macrophages through chemokine axes such as CCL2-CCR2, facilitating clearance of inflammatory cells after CNS insults like ischemic stroke (106). This trafficking is critical for modulating inflammation and tissue repair. Furthermore, the meninges harbor specialized immune niches, including dural-associated lymphoid tissues (DALT) that support B cell development and humoral immune responses to systemic and nasal antigens and local pathogen challenge (107). Specialized barrier cells within the choroid plexus act as a barrier to control communication between the periphery and the CNS, maintaining compartmentalization of the choroid plexus, brain parenchyma and CSF while allowing controlled immune communication (108).

This immune crosstalk between the meninges and brain parenchyma has significant implications for neuroinflammatory and neurodegenerative diseases. Dysfunction or remodeling of meningeal lymphatic vessels, often observed with aging or in CNS disorders, can impair CSF drainage, leading to accumulation of toxic proteins and altered immune surveillance that exacerbate disease progression (109, 110). Moreover, the meningeal immune environment influences the recruitment and activation of peripheral immune cells that infiltrate the CNS during pathological conditions such as multiple sclerosis, providing novel targets for immunotherapeutic interventions (111). The meningeal compartment thus represents a critical hub where immune signals are integrated and relayed, bridging the peripheral immune system and brain parenchyma.

In summary, the immune communication between the meninges and brain parenchyma is orchestrated through a complex interplay of cytokines, chemokines, and lymphatic drainage pathways. Immune cells traffic via meningeal lymphatics into the CNS, participate in inflammatory responses and immune surveillance, and contribute to the pathophysiology of CNS diseases. Targeting these communication mechanisms offers promising avenues for novel immunotherapies in neurological disorders. Given the emerging evidence, it is plausible that enhancing meningeal lymphatic function or modulating meningeal immune cell activity could improve CNS immune homeostasis and therapeutic outcomes in glioblastoma and other neuroinflammatory conditions (104, 112).

### 3.4 Potential role of the meningeal immune system in tumor immunity

The meningeal immune system has emerged as a critical frontier in understanding central nervous system immunity, particularly in the context of brain tumors such as glioblastoma. Traditionally, the CNS was considered an immune-privileged site due to the presence of the blood-brain barrier and the absence of classical lymphatic drainage. However, the recent discovery of functional meningeal lymphatic vessels has challenged this notion, revealing an active interface between the CNS and the peripheral immune system (113). MLVs serve as conduits for cerebrospinal fluid drainage and facilitate the transport of CNS-derived antigens and immune cells to deep cervical lymph nodes, thereby acting as a sentinel system that primes immune surveillance against brain tumors.

In glioblastoma, the meningeal immune system may function as a frontline regulator of anti-tumor immunity. Resident border-associated macrophages within the dura mater have been characterized by high neonatal Fc receptor expression, which endows them with superior antigen-presenting capacities. These rBAMs can activate CNS-patrolling T cells, amplifying cytotoxic T cell responses within the tumor microenvironment (15). Notably, experimental blockage of meningeal blood vessels suppresses GBM progression, highlighting the functional importance of meningeal immune components in tumor control. This suggests that the meningeal immune system is not merely a passive barrier but an active modulator that orchestrates the initiation and amplification of tumor-specific immune responses.

Therapeutic modulation of the meningeal immune environment holds promise for overcoming the immunosuppressive milieu characteristic of GBM. For instance, enhancing meningeal lymphatic drainage through vascular endothelial growth factor C (VEGF-C) administration leads to potent priming of CD8+ T cells and effective tumor eradication in preclinical glioblastoma models (114, 115). Such strategies effectively convert immunologically “cold” tumors into “hot” tumors, thereby improving immune recognition and attack. Furthermore, meningeal lymphatic dysfunction, which can occur with aging or tumor progression, correlates with reduced immune cell trafficking and impaired tumor clearance, indicating that restoring meningeal lymphatic function may enhance immunosurveillance (116).

**Figure 2:**
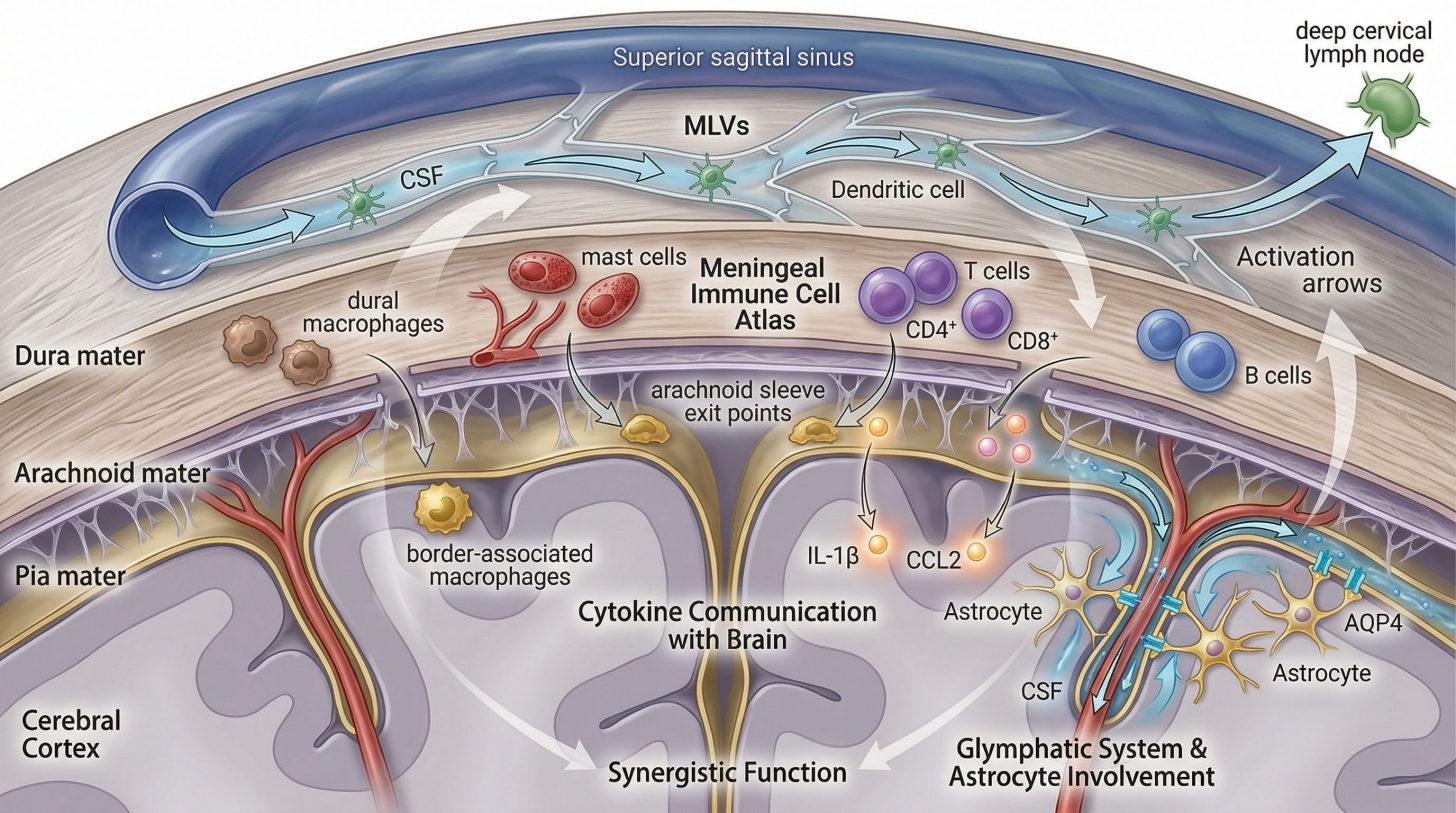
Anatomical and functional basis of the meningeal immune system.

The meningeal immune system also represents a novel therapeutic target to surmount the challenges posed by the blood-brain barrier and the immunosuppressive tumor microenvironment. By regulating antigen drainage and immune cell activation at the meningeal interface, it is possible to improve the delivery and efficacy of immunotherapies for GBM (117). Additionally, meningeal lymphatics facilitate communication between intracranial and peripheral immunity, suggesting that interventions targeting this pathway could synergize with systemic immune-modulating treatments. This integrated approach may help to break the cycle of immune evasion and resistance that currently limits the success of GBM immunotherapy (**Figure 2**).

In summary, the meningeal immune system acts as a crucial immune sentinel and regulator in brain tumor immunity. Its capacity to modulate antigen presentation, T cell activation, and immune cell trafficking provides a mechanistic basis for novel immunotherapeutic strategies aimed at enhancing anti-GBM immunity. Targeting the meningeal immune environment, particularly through modulation of meningeal lymphatic function, offers a promising avenue to overcome current therapeutic barriers and improve clinical outcomes in glioblastoma patients (62).

## 4 Discovery and mechanism of rBAM cells

### 4.1 Identification of resident border-associated macrophages

Resident border-associated macrophages are a distinct population of macrophages localized specifically at the brain’s meningeal boundaries, including the dura mater, perivascular spaces, and choroid plexus. Unlike parenchymal microglia, these rBAMs occupy the interface between the central nervous system and the peripheral immune environment, positioning them strategically to regulate immune surveillance and homeostasis at this critical border zone. Their unique localization is accompanied by a specialized phenotype and gene expression profile that distinguish them from traditional brain-resident microglia and circulating monocyte-derived macrophages. Recent advances employing single-cell RNA sequencing (scRNA-seq) and spatial transcriptomics have been instrumental in delineating these differences, revealing that rBAMs express high levels of neonatal Fc receptor (FcRn), which is less prominent in other CNS macrophage subsets (15).

The characterization of rBAMs has been greatly facilitated by combining single-cell transcriptomic approaches with immunohistochemical validation. Single-cell RNA sequencing in mouse models has revealed that rBAMs, microglia, and infiltrating monocytes/macrophages have distinct transcriptional profiles (118). Immunohistochemistry further confirms their meningeal localization, and specific markers such as CD206 and CD163 have been used to identify rBAMs *in situ*. These studies underscore the fact that rBAMs are not merely passive bystanders but active participants in CNS immune regulation, particularly at the meningeal borders where they sample CNS-derived antigens and interact with patrolling T cells (15).

Functionally, rBAMs contribute significantly to both immune homeostasis and pathological responses in the CNS. Under steady-state conditions, they maintain meningeal immune surveillance by presenting CNS antigens and supporting T cell activation, thus forming a crucial line of defense against CNS infections and malignancies. In pathological states such as glioblastoma or ischemic stroke, rBAMs undergo dynamic transcriptional reprogramming. For example, in glioblastoma, rBAMs have been shown to expand and enhance anti-tumor immunity by promoting cytotoxic T cell responses, which correlates positively with patient survival (15). Conversely, after ischemic stroke, rBAMs activate inflammatory pathways, including the tumor necrosis factor signaling cascade and Stat3 transcription factor activity, which can exacerbate neuroinflammation and tissue damage (119). These functional dualities highlight the plasticity of rBAMs in responding to CNS insults.

Interestingly, the expansion and activation of rBAMs can be modulated by alterations in meningeal blood flow and cytokine milieu. Experimental models demonstrate that blockage of meningeal blood vessels preserves CSF-1 levels in the dura mater, thereby restricting the influx of circulating macrophages and selectively expanding the rBAM population. This expansion enhances T cell activation at the dura interface and amplifies intratumoral cytotoxic responses against glioblastoma, suggesting a potential therapeutic avenue to potentiate immunotherapy by targeting rBAMs (15). Such findings imply that rBAMs might serve as a critical immunological checkpoint within the meningeal niche, balancing immune activation and suppression in CNS diseases.

Taken together, the identification and characterization of rBAMs as a unique macrophage subset residing at CNS borders with distinct phenotypic and functional properties provide new insights into meningeal immunity. Their role in maintaining immune homeostasis and modulating disease progression, particularly in glioblastoma and neuroinflammatory conditions, opens promising directions for therapeutic strategies aiming to harness or modulate rBAM activity. Further research is warranted to elucidate the molecular mechanisms governing rBAM differentiation, maintenance, and crosstalk with other immune cells, which may reveal novel targets for improving CNS disease outcomes (15, 119, 120).

### 4.2 rBAM’s antigen presentation function

Resident border-associated macrophages in the meninges have emerged as pivotal players in central nervous system immunosurveillance, particularly in the context of glioblastoma. These macrophages exhibit a high capacity for antigen uptake and presentation, enabling them to efficiently activate T cell-mediated immune responses. Single-cell profiling studies have identified a subset of rBAMs characterized by elevated expression of the neonatal Fc receptor, which confers superior ability to present tumor antigens to CNS-patrolling T cells (15). This antigen presentation capability is critical in the GBM microenvironment, where rBAMs facilitate the initiation of tumor-specific immune responses by processing and presenting tumor-derived antigens, thereby promoting the activation and proliferation of cytotoxic T lymphocytes within the dura mater. The expansion of the rBAM population, for example through meningeal blood vessel blockage, has been shown to enhance T cell activation at the dura interface and amplify intratumoral cytotoxic T cell responses, correlating positively with improved patient survival (15). These findings underscore the importance of rBAMs as immunological sentinels that bridge innate and adaptive immunity in GBM.

In addition to their direct antigen-presenting function, rBAMs interact synergistically with other immune cell types to strengthen the immune system’s ability to recognize and respond to tumor cells. Mural cells in the dura mater physically contact macrophages and modulate their activity; specifically, mural cells can transfer cytoplasmic components including RNA granules that can reprogram macrophage transcriptomes (121). This crosstalk suppresses excessive antigen-dependent T helper cell activation and differentiation, thus fine-tuning the immune response to prevent overactivation while maintaining effective surveillance. Moreover, in neuroinflammatory conditions, combined depletion of both microglia and meningeal macrophages, including rBAMs, leads to reduced expression of major histocompatibility complex II (MHC II) and co-stimulatory molecules such as CD80 on antigen-presenting cells, resulting in diminished T cell reactivation and proliferation (122). These observations highlight that rBAMs not only present antigens but also coordinate with other immune cells to sustain a balanced yet potent anti-tumor immunity in the CNS border regions.

The unique anatomical localization of rBAMs at the CNS borders allows them to serve as critical gatekeepers for immune cell trafficking and antigen presentation. Their strategic position in the dura mater enables them to sample CNS-derived antigens and present them to patrolling T cells, effectively linking peripheral immune surveillance with central immune responses (15). This spatial advantage is essential in GBM, where the immunosuppressive tumor microenvironment often hampers effective immune activation. By promoting tumor-specific immune responses at the meningeal interface, rBAMs contribute to overcoming local immune evasion mechanisms. Furthermore, the abundance of rBAMs correlates with patient prognosis, suggesting that therapeutic strategies aimed at expanding or enhancing rBAM function could potentiate immunotherapy efficacy in GBM. For instance, surgical meningeal vascular blockage preserves dural colony-stimulating factor 1 (CSF-1) levels, which can expand the rBAM pool and improve T cell-mediated anti-glioblastoma activity (15). This provides a rationale for targeting rBAMs as a novel immunotherapeutic avenue.

Collectively, the evidence supports that rBAMs possess robust antigen uptake and presentation capabilities that are indispensable for eliciting effective T cell responses against GBM. Their interactions with other immune components and their localization at the CNS boundaries position them as key orchestrators of tumor-specific immunity within the meningeal microenvironment. Enhancing rBAM function or numbers may represent a promising strategy to amplify anti-tumor immunity and improve outcomes for patients with glioblastoma. Future research should focus on elucidating the molecular mechanisms governing rBAM antigen presentation and their crosstalk with other immune cells to optimize immunotherapeutic interventions targeting these macrophages.

### 4.3 rBAM-mediated immune regulatory mechanisms

Resident border-associated macrophages in the dura mater play a crucial role in modulating the local immune microenvironment through their secretion of cytokines and chemokines, which facilitate the recruitment and activation of immune cells. The dura mater, as the outermost meningeal layer, acts as an essential interface for central nervous system immunosurveillance by sampling and presenting CNS-derived antigens. Recent single-cell profiling studies have identified a subset of rBAMs characterized by high expression of the neonatal Fc receptor, endowing them with an enhanced ability to present tumor antigens and activate CNS-patrolling T cells. This antigen-presenting capacity allows rBAMs to orchestrate robust anti-tumor immune responses within the meningeal compartment, particularly in glioblastoma models where rBAM abundance correlates positively with patient survival (15). The secretion of specific cytokines and chemokines by rBAMs establishes a chemotactic gradient that recruits effector immune cells such as cytotoxic T lymphocytes, thereby amplifying intratumoral cytotoxic responses. This dynamic underscores the pivotal role of rBAMs in sculpting the immune landscape at the CNS borders.

The immune regulatory functions of rBAMs are profoundly influenced by the tumor microenvironment, which can modulate their phenotype toward either immunosuppressive or immunostimulatory states. Within the GBM microenvironment, factors such as colony-stimulating factor 1 levels and meningeal blood vessel integrity impact the balance between circulating BAMs (cBAMs) and rBAMs. Meningeal blood vessel blockage preserves dural CSF-1, which favors the expansion of the rBAM pool and enhances T cell activation at the dura interface. Conversely, the TME can induce immunosuppressive programs within rBAMs, potentially dampening anti-tumor immunity. rBAMs promote anti-tumor immune activation by presenting tumor antigens and activating T cells against glioblastoma (15). Such plasticity highlights the complexity of rBAM-mediated immune regulation and the necessity to understand the specific molecular cues governing their function in glioma progression.

Given their central role in shaping local immune responses, rBAMs represent promising therapeutic targets for immunomodulation in glioblastoma. Strategies aiming to manipulate rBAM function—such as enhancing their antigen-presenting capacity or modulating their cytokine secretion profile—could potentiate anti-tumor immunity. For instance, interventions that preserve or augment rBAM populations, possibly via meningeal blood vessel modulation, have demonstrated efficacy in enhancing T cell activation and improving tumor control in preclinical models (15). Moreover, targeting the signaling pathways and receptors involved in rBAM activation and recruitment may provide additional avenues to overcome tumor-induced immunosuppression. The identification of rBAM-specific markers and their functional characterization lays the groundwork for the development of novel immunotherapeutic approaches that leverage the unique immune regulatory capabilities of these macrophages in the CNS border regions.

In summary, rBAMs mediate immune regulation in the meningeal environment through cytokine and chemokine secretion, facilitating immune cell recruitment and activation. Their functions are modulated by the tumor microenvironment, which can tip the balance toward immune activation or suppression. Targeting rBAMs and their regulatory mechanisms holds significant potential for enhancing immunotherapeutic efficacy against glioblastoma. The intricate interplay between rBAMs, the meningeal vasculature, and tumor-derived factors suggests that combinatorial strategies addressing multiple components of this axis may yield the most effective clinical outcomes.

### 4.4 MBB intervention strategies and their mechanisms of action

The meningeal border blockade (MBB) strategy represents a novel immunotherapeutic approach targeting glioblastoma by modulating the function of resident border-associated macrophages at the dura mater, the outermost meningeal layer. This strategy enhances the immune system’s ability to attack tumor cells localized at the brain tumor boundaries. Recent studies have identified a specialized subset of rBAMs characterized by high expression of neonatal Fc receptor, which endows them with superior antigen-presenting capabilities and the ability to activate central nervous system -patrolling T cells effectively. By blocking meningeal blood vessels, the MBB approach preserves colony-stimulating factor 1 levels in the dura, which restricts the infiltration of circulation-derived BAMs and selectively expands the rBAM pool. This expansion leads to heightened T cell activation at the dura interface and amplifies the cytotoxic T cell responses within the tumor microenvironment, thereby potentiating anti-glioblastoma immunity. Clinically, higher rBAM abundance has been correlated with improved survival outcomes in GBM patients, underscoring the therapeutic potential of MBB (15).

Mechanistically, MBB facilitates immune cell infiltration by modulating the meningeal microenvironment, which serves as a critical immunological interface between the CNS and peripheral immunity. The dura mater hosts diverse immune cell populations and functions as a surveillance site for CNS-derived antigens. By enhancing rBAM function and maintaining a favorable cytokine milieu, MBB promotes the recruitment and activation of effector immune cells, including cytotoxic CD8+ T lymphocytes, at the tumor margin. This enhanced immune surveillance is crucial given the immunosuppressive nature of the GBM microenvironment and the restrictive blood-brain barrier that limits immune cell trafficking. The MBB strategy thereby enhances anti-glioblastoma immunity and has been proposed as a surgical strategy to improve the efficacy of immunotherapy in GBM (15).

Furthermore, MBB intervention has been shown to improve the permeability of meningeal barriers, facilitating immune cell transmigration from the periphery into the CNS. This is particularly important because the meningeal lymphatic vessels and associated immune networks serve as conduits for antigen drainage and immune cell trafficking. Enhancing the function of these meningeal immune gateways can increase the infiltration of adoptively transferred or endogenous immune cells into glioblastoma sites. For example, adoptively transferred innate lymphoid cells type 2 (ILC2s) have been demonstrated to localize within intracranial glioblastoma tumors and meninges, and direct tumor growth inhibition was not observed in the initial study (123). This indicates that MBB may act synergistically with other immunomodulatory therapies to achieve significant therapeutic effects.

Importantly, MBB provides a theoretical and technical foundation for “Interstitial” immunotherapy paradigms. By transiently modulating the meningeal immune environment and barrier functions, MBB allows periodic enhancement of immune cell access and activation without continuous disruption of CNS homeostasis. This Interstitial modulation could reduce immune-related adverse events and improve the sustainability of immune responses against GBM. The concept aligns with emerging insights into the glymphatic-immune axis, where controlled manipulation of fluid and immune cell dynamics at CNS borders enhances immunosurveillance and therapeutic delivery (124). Thus, MBB stands as a promising adjunct to existing immunotherapies, potentially enabling more effective and safer treatment regimens through controlled, time-spaced immune activation.

**Figure 3:**
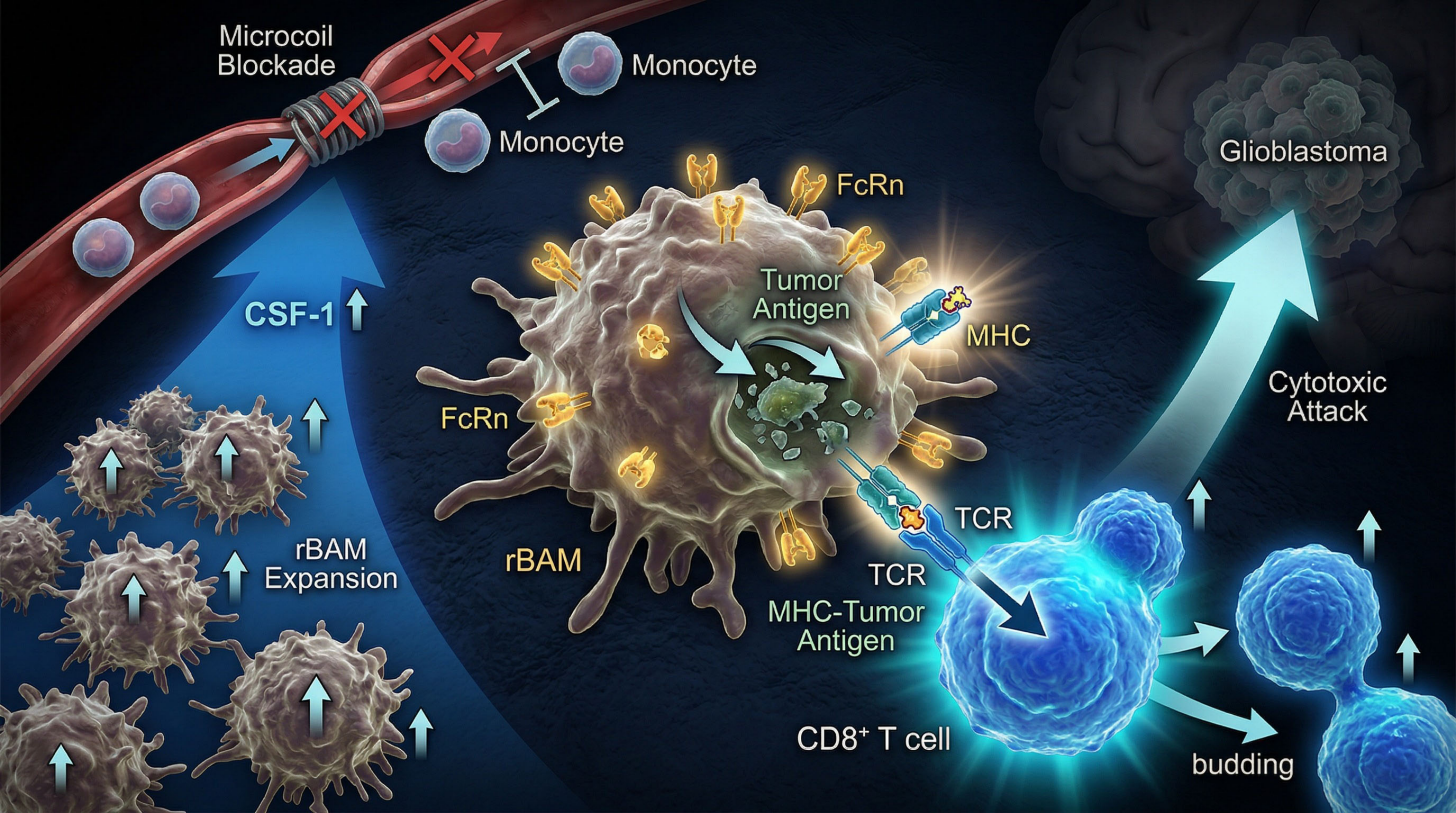
Meningeal blood vessel blockage (MBB) expands rBAMs and activates anti−GBM T cell immunity.

In summary, MBB intervention strategies harness the unique immunological properties of the meningeal border by regulating rBAM function, promoting immune cell trafficking across meningeal barriers, and enhancing tumor-specific immune responses. These effects collectively improve the efficacy of glioblastoma immunotherapy and provide a mechanistic basis for developing Interstitial treatment protocols that optimize immune activation while minimizing toxicity. Future research should focus on integrating MBB with nanotechnology-based delivery systems and lymphatic-targeted therapies to further potentiate immune responses and overcome the formidable challenges presented by the GBM tumor microenvironment (114, 125) (**Figure 3**).

## 5 The connotation of “Interstitial” immunotherapy

### 5.1 “Interstitial” therapy concept analysis

The concept of “Interstitial” therapy in glioblastoma treatment represents a novel paradigm that emphasizes targeting the tumor boundary region rather than focusing solely on the tumor core. This approach leverages the unique advantages of the meningeal immune system, particularly the meningeal lymphatic vessels, to establish an immunological barrier that can prevent tumor cells from invading the brain parenchyma. Unlike traditional therapies that concentrate on direct cytotoxic effects within the tumor mass, interstitial immunotherapy targeting the meningeal border region aims to modulate the immune microenvironment at the tumor margins, creating a dynamic immune defense line that can intercept and eliminate migrating tumor cells before they colonize deeper brain tissue.

The meningeal lymphatic system has emerged as a critical interface for central nervous system immunosurveillance. It facilitates the drainage of cerebrospinal fluid and CNS-derived antigens to peripheral lymph nodes, enabling immune cell priming and activation. By targeting the tumor border zone, interstitial therapy seeks to harness this pathway to enhance antigen presentation and T cell activation in the draining deep cervical lymph nodes, thereby amplifying anti-tumor immune responses. VEGF-C-mediated enhancement of meningeal lymphatic function enhances immune surveillance of brain tumors and synergizes with immune checkpoint blockade to eradicate glioblastoma in preclinical models (114, 115).

Interstitial therapy also distinguishes itself by addressing the challenge of glioblastoma’s invasive nature. Tumor cells frequently infiltrate adjacent brain tissue beyond the visible tumor margin, evading localized treatments such as surgery and radiotherapy. By focusing on the tumor boundary and its immediate microenvironment, interstitial therapy aims to disrupt the migratory pathways and immunosuppressive niches that facilitate tumor spread. This may involve enhancing the function of resident border-associated macrophages in the dura mater, which have been shown to possess superior antigen-presenting capabilities and contribute to the activation of CNS-patrolling T cells, thus reinforcing the immune barrier at the tumor edge (91).

In summary, the interstitial therapy concept represents an innovative shift from traditional glioblastoma treatment paradigms by exploiting the meningeal immune system’s unique properties to establish an immunological defense at the tumor border. This approach not only aims to block tumor cell dissemination into the brain parenchyma but also to synergize with systemic immunotherapies, potentially improving clinical outcomes. Future research focusing on meningeal immune activation could investigate integrating strategies with emerging modalities such as photodynamic therapy and sonodynamic therapy to maximize therapeutic efficacy (126).

### 5.2 Immune cell activation targeting the tumor border

Activation of immune cells at the tumor border represents a pivotal strategy in enhancing local anti-glioblastoma immune responses, leveraging the unique immunological environment of the meninges. Resident border-associated macrophages, a specialized subset of meningeal immune cells, have been identified as key players in this context due to their superior antigen-presenting capabilities and capacity to activate CNS-patrolling T cells. Recent single-cell profiling studies of the dura mater revealed that rBAMs express high levels of neonatal Fc receptor, which equips them to effectively present tumor-derived antigens and promote effector T cell activation near the tumor boundary. Notably, experimental meningeal blood vessel blockage expands the rBAM pool by preserving colony-stimulating factor 1 levels, thereby amplifying T cell responses both at the dura interface and within the tumor microenvironment. This spatially targeted immune activation at the tumor border correlates with improved glioblastoma patient survival, suggesting that strategies harnessing rBAM-mediated T cell activation could potentiate local tumor clearance (15).

In addition to rBAMs, the tumor border is characterized by a complex immunological milieu involving various myeloid and lymphoid populations. Quantitative immunohistochemical analyses of glioblastoma margins have demonstrated that anti-inflammatory microglia and macrophages expressing markers such as triggering receptor expressed on myeloid cells 2 (TREM2), CD163, and CD32a are enriched at the invasive edge. These cells correlate positively with CD8+ T cell infiltration and programmed death-ligand 1 (PD-L1) expression, indicating an immune-suppressive yet dynamic interface where immune cells congregate and interact. The co-localization of activated cytotoxic T cells with immunosuppressive myeloid cells suggests a tightly regulated immune microenvironment that could be modulated to enhance anti-tumor immunity. Importantly, high expression of these myeloid markers at the tumor border is associated with poorer overall survival, highlighting the need to shift the balance toward effective immune activation rather than suppression in this region (127). This interplay underscores the potential of targeting tumor border immune cells to disrupt immunosuppressive networks and reinvigorate T cell-mediated tumor eradication.

interstitial therapeutic approaches may optimize immune cell delivery and sustained activation at the tumor border. Post-surgical dynamics of the tumor microenvironment reveal a transient opening of the blood-brain barrier and evolving immune cell spatial distribution over time. Studies employing syngeneic mouse models of glioblastoma resection have shown that the early post-surgical period is marked by the accumulation of reactive microglia and anti-inflammatory macrophages at the resection cavity borders, which can promote tumor recurrence. Combining localized nanomedicine hydrogels with systemic immunomodulatory agents, administered interstitial post-surgery, has demonstrated enhanced microglial anti-tumoral activity, reduced immunosuppressive macrophages, and delayed tumor regrowth. Postoperative GBM microenvironment exhibits time-dependent immune responses, and combination therapy targeting this process can reverse the immune landscape and delay glioblastoma recurrence (128). Such strategies could be further refined by leveraging the unique properties of meningeal immune cells and their interactions with infiltrating lymphocytes.

The spatial distribution of immune activation at the tumor border also correlates with imaging features indicative of immune activity. MRI analyses have linked thicker contrast-enhancing tumor margins with increased infiltration of activated cytotoxic CD8+ T cells and central memory T cells, suggesting that radiographic features may serve as non-invasive biomarkers of immune cell localization and activation. This correlation supports the notion that immune cell clustering at the tumor periphery is not only a histological phenomenon but also manifests in imaging characteristics that could guide patient stratification and treatment planning. Furthermore, the major immune cell population at peri-necrotic rims of GBM is neutrophils, indicating ongoing immune responses at tumor boundaries that may be harnessed or augmented therapeutically (129). These insights provide a rationale for integrating imaging and immunology to monitor and optimize immune activation at the glioblastoma margin.

Collectively, these findings emphasize the critical role of immune cell activation at the glioblastoma tumor border, driven by meningeal immune populations such as rBAMs and modulated by the balance of pro- and anti-inflammatory myeloid cells. Interstitial therapeutic regimens that enhance effector T cell activation and sustain immune cell presence at the tumor margin hold promise for improving local tumor control and patient outcomes. Future research should focus on elucidating the molecular signals governing immune cell recruitment and activation at this interface, as well as developing strategies to exploit these mechanisms for durable immunotherapeutic effects in glioblastoma.

### 5.3 Remodeling of the immune microenvironment and reversal of immune tolerance

The concept of “interstitial” therapy in glioblastoma immunotherapy hinges significantly on its ability to modulate the meningeal immune environment, thereby mitigating the expression of immunosuppressive factors. Combined blockade of TGF-β and PD-L1 pathways has been shown to induce potent anti-tumor immune responses against glioma (130). This modulation of the meningeal immune milieu is critical, as the meninges serve as an immunological interface that influences CNS immune surveillance and tumor immunity (62). By transiently disrupting the immunosuppressive signaling within the meninges, interstitial therapy can promote the recruitment and activation of effector immune cells, including cytotoxic CD8+ T cells and pro-inflammatory macrophages, thereby enhancing anti-tumor immunity.

The remodeling of the immune microenvironment at the tumor border is another pivotal aspect of interstitial therapy. Elevated M2 macrophage infiltration in GBM is correlated with immune checkpoint activation (131). Cisplatin treatment can promote TAMs toward M1-like polarization and enhance pro-inflammatory activation of TAMs, and combination therapy with DFMO, aPD-L1 and photothermal therapy can induce TAM phenotype switching and enhance phagocytic activity (132) (133). This reprogramming alleviates immune tolerance at the tumor margins, facilitating the infiltration and function of cytotoxic lymphocytes. Moreover, cisplatin treatment can downregulate immunosuppressive molecules such as CD47-SIRPα axis components, thereby promoting M1-like polarization of tumor-associated macrophages and enhancing anti-tumor immune activation in the microenvironment (132). The dynamic reshaping of the tumor border microenvironment thus serves to restore immune surveillance and break the local immune tolerance that typically favors tumor progression.

This process of immune microenvironment remodeling and tolerance reversal involves the coordinated regulation of multiple cytokines and signaling pathways. Key cytokines implicated include interferon-γ (IFN-γ), which enhances antigen presentation and T cell activation, and chemokine CXCL10, which recruits CD8+ tumor-infiltrating lymphocytes into the tumor microenvironment (134). Signaling pathways such as the PI3K/AKT/mTOR axis are modulated to suppress tumor cell proliferation and promote immune activation (135). Additionally, combined blockade of immunosuppressive pathways including TGF-β and PD-L1 has been shown to synergistically enhance anti-tumor immune responses against glioma (130). The interplay between these molecular mediators orchestrates a shift from an immunosuppressive to an immunostimulatory milieu, facilitating durable anti-tumor immunity. Notably, VEGF-C-mediated expansion of meningeal lymphatic vessels imsuggests antigen drainage and T cell priming in deep cervical lymph nodes, further potentiating systemic immune responses against glioblastoma (114). Collectively, these multifaceted regulatory mechanisms underscore the potential of interstitial therapeutic strategies to comprehensively reprogram the GBM immune microenvironment and overcome intrinsic immune tolerance.

**Figure 4:**
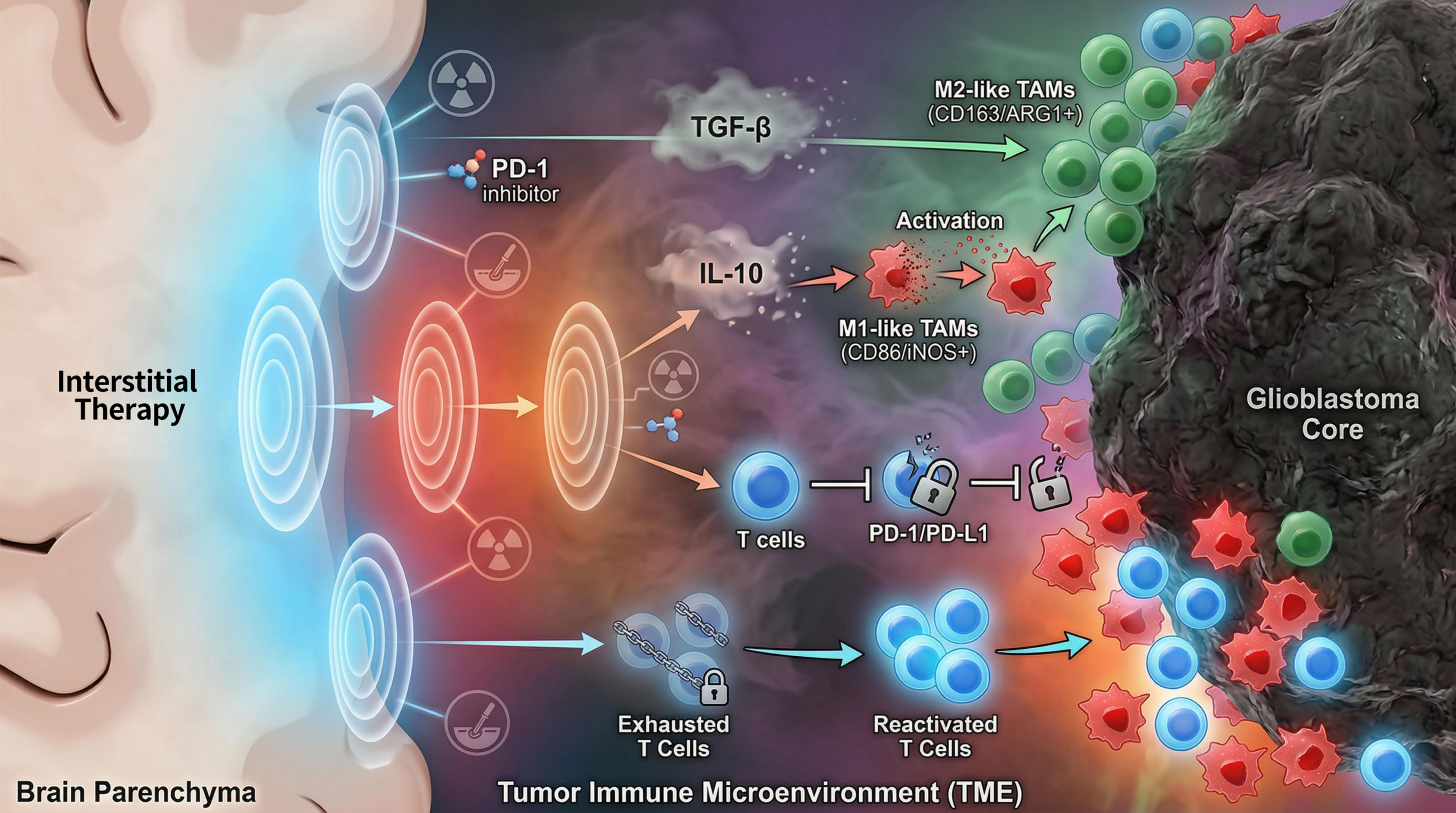
Interstitial therapy reprograms the GBM tumor immune microenvironment and reverses immune tolerance.

In summary, interstitial therapy exerts its therapeutic benefit in glioblastoma by remodeling the meningeal and tumor border immune microenvironments, reducing immunosuppressive factor expression, and reversing immune tolerance. This is achieved through the coordinated action of cytokines and signaling pathways that promote pro-inflammatory macrophage polarization, T cell infiltration, and activation. Such immune microenvironment reprogramming represents a promising paradigm to enhance the efficacy of immunotherapy in GBM, warranting further clinical investigation and optimization (**Figure 4**).

### 5.4 Advantages and limitations of “Interstitial” therapy

Unlike systemic dosing, interstitial schedules can achieve higher peak concentrations of drugs or immunomodulatory agents within the tumor microenvironment, thereby enhancing local bioavailability without causing sustained systemic toxicity (136). For example, high-dose administration of targeted agents like sunitinib was designed to increase intratumoral drug accumulation, but clinical trials found that neither of the tested high-dose sunitinib regimens improved progression-free survival compared to lomustine in recurrent glioblastoma, and the trial was terminated early due to lack of efficacy (137). Additionally, the potential for therapy to induce resistance mechanisms, such as mesenchymal-to-epithelial transition observed in temozolomide-resistant glioblastoma cells, warrants caution (138). Therefore, the principle of interstitial dosing remains attractive as it may transiently disrupt BBB integrity or exploit windows of enhanced permeability, facilitating immune cell infiltration and drug penetration.

Despite these advantages, interstitial therapy faces several limitations primarily related to the complexity of the meningeal immune system and technical challenges in treatment delivery. The meningeal immune environment is highly specialized and dynamically regulated, involving interactions among lymphatic vessels, resident macrophages, dendritic cells, and T cells. This complexity implies that interstitial interventions may have unpredictable effects on immune cell trafficking and function. For example, the heterogeneity of blood vessel permeability and interstitial blood flow within GBM tumors can affect drug delivery to tumor cells (139). Furthermore, the technical difficulty of precisely controlling interstitial dosing schedules, particularly for locoregional therapies such as convection-enhanced delivery (CED), remains a barrier. Studies in agarose gel brain models have shown that catheter movement protocols during CED significantly influence drug dispersal volumes and distribution patterns (140).

Another limitation is the current lack of comprehensive safety and efficacy data from large clinical trials. While early-phase studies suggest feasibility and safety, the optimal parameters for interstitial therapy—such as dose intensity, frequency, and duration—require further refinement. For instance, interstitial sonodynamic therapy combined with temozolomide showed tolerability but did not demonstrate clear long-term survival benefits in a small patient cohort, highlighting the need for larger, controlled studies (141). Tailoring interstitial regimens to individual tumor biology and immune status may be necessary to overcome these challenges.

In conclusion, interstitial therapy offers several compelling advantages for glioblastoma treatment by enhancing drug delivery across the BBB, promoting immune cell infiltration, and reducing systemic toxicity. However, these benefits are tempered by the intricate biology of the meningeal immune system and technical hurdles in therapy administration. Continued efforts to optimize treatment protocols and rigorously evaluate safety and efficacy are essential to fully harness the potential of interstitial immunotherapy in glioblastoma.

## 6 Clinical translation prospects and challenges

### 6.1 Operability analysis of meningeal vascular embolization

While preclinical rationale supports meningeal vascular embolization as a plausible strategy to modulate the meningeal immune milieu in neuro-oncological contexts like glioblastoma, clinical evidence regarding its feasibility remains limited. Although advancements in neuroendovascular instrumentation theoretically enable precise catheter navigation and targeted occlusion, current data are largely extrapolated from broader neurointerventional experiences; for instance, specialized distal access guide catheters such as the SelectFlex system have demonstrated 100% success in accessing target cerebral vasculature across diverse procedures, including middle meningeal artery (MMA) embolization, with a low rate of serious adverse events (0.3%) (142). Consequently, while existing endovascular technology suggests that meningeal vascular embolization could serve as a practical tool for manipulating the meningeal immune environment, explicit clinical validation specific to this immunomodulatory application is still required to distinguish hypothesis from established therapeutic reality.

From a safety perspective, the procedure benefits from improvements in embolic materials and delivery methods. The SwiftPAC coil system, characterized by its innovative two-dimensional wave-like design, has been shown to achieve precise vessel occlusion in cerebrovascular pathologies with minimal complications (143). Additionally, the isolated use of coils for MMA embolization has been reported to be as effective as combined coil and particle embolization, while reducing risks related to cranial nerve and visual complications associated with particle embolization (144). These findings underscore the safety profile of meningeal vascular embolization when performed with modern materials and techniques. However, caution is warranted due to potential complications such as inadvertent embolization of collateral vessels; for example, embolization of the middle meningeal artery has been associated with rare but severe complications like central retinal artery occlusion, emphasizing the need for meticulous pre-procedural vascular mapping and intraoperative monitoring (145).

Mechanistically, meningeal vascular embolization modulates immune cell trafficking and the immunological landscape at the tumor margin by altering meningeal hemodynamics and vascular permeability. As the meningeal vasculature constitutes a pivotal gateway for immune infiltration into the central nervous system and the tumor microenvironment, occluding these arteries can disrupt pathological neovascularization and curtail oxygen and nutrient delivery, thereby reshaping the local immune milieu. Preclinical investigations in chronic subdural hematoma (CSDH) have demonstrated that pathological sinusoidal capillaries within the dura mater and outer membranes are perfused by the middle meningeal arteries; consequently, embolizing these vessels effectively ablates blood flow to such aberrant structures, representing a validated therapeutic strategy for CSDH (146). By analogy, while preclinical evidence suggests that targeted embolization in glioblastoma could similarly obstruct the aberrant meningeal vasculature sustaining immunosuppressive niches—potentially augmenting immune cell infiltration and activation at the tumor boundary—clinical evidence supporting this extrapolation remains limited.

Furthermore, meningeal vascular embolization may modulate immune cell trafficking by reshaping chemokine gradients and altering the expression of vascular adhesion molecules. The mechanical and biochemical perturbations resulting from vessel occlusion could theoretically favor the recruitment of effector immune cells while impeding the migration of immunosuppressive populations. Although direct clinical evidence in glioblastoma remains scarce, preclinical data establishing the critical role of meningeal lymphatics and vasculature in central nervous system immune surveillance suggest that manipulating meningeal blood flow has the potential to reconfigure the tumor-immune interface. preclinical evidence suggests that embolization-induced hypoperfusion and vascular remodeling might disrupt the immunosuppressive microenvironment to enhance antigen presentation and T-cell activation; however, this hypothesis requires rigorous validation before it can be considered a viable strategy to synergize with immunotherapies.

In clinical practice, rigorous risk mitigation and surveillance during meningeal vascular embolization are indispensable for safeguarding patient well-being and procedural success. Hazards encompass ischemic sequelae arising from inadvertent vessel occlusion, hemorrhagic incidents, and the displacement of embolic agents into non-target territories. Countermeasures include exhaustive preoperative angiographic mapping to define vascular architecture and collateral routes, alongside balloon protection strategies to maintain critical venous outflow during embolization (147). Moreover, employing manual compression to arrest flow from cutaneous feeders has been demonstrated to improve embolic penetration while curtailing adverse events (148, 149). Post-procedural imaging and clinical monitoring remain vital for the early identification of complications such as vasospasm or ischemia. Collectively, these risk management frameworks enable the secure deployment of meningeal vascular embolization within intricate neuro-oncological contexts.

Synthesizing current findings, meningeal vascular embolization appears to be a technically viable and safe strategy for modulating the meningeal immune milieu, with proposed mechanistic impacts on immune cell trafficking and tumor boundary immunity. While procedural risks can be effectively governed through comprehensive preoperative planning and intraoperative oversight, it is crucial to distinguish between established facts and theoretical projections. Preclinical evidence suggests that this modality could serve as a pivotal component of “interstitial” therapeutic paradigms aimed at bolstering glioblastoma immunotherapy by reshaping the tumor-immune interface via vascular modulation; however, clinical evidence remains limited regarding its definitive immunological consequences in patients. Consequently, further clinical investigations are warranted to refine embolization protocols and clearly elucidate the immunological outcomes in the glioblastoma population.

### 6.2 Potential of combination with immune checkpoint inhibitors

The combination of interstitial therapeutic strategies with immune checkpoint inhibitors holds significant promise in enhancing the immune activation against glioblastoma. Interstitial treatments can induce immunogenic cell death and modulate the tumor microenvironment, thereby priming it for improved immune responses. ICIs, which target inhibitory pathways like PD-1/PD-L1, can then relieve the immune suppression imposed by the tumor, allowing T cells and other immune effectors to mount a more effective antitumor response. For instance, preclinical evidence suggests that agents capable of reprogramming tumor-associated macrophages from an immunosuppressive M2 phenotype to a pro-inflammatory M1 state can sensitize GBM to ICI therapy, as demonstrated by the synergy between rapamycin, hydroxychloroquine, and anti-PD-1 treatment in murine models (150). However, clinical evidence remains limited; while these data indicate that interstitial regimens altering the immune architecture may synergize with checkpoint blockade to overcome the profound immunosuppression characteristic of GBM, the translation of this plausible biological logic into established clinical feasibility requires further validation to clearly distinguish current experimental support from therapeutic certainty.

The mechanism by which ICIs exert their effects involves the disruption of immune checkpoint pathways that tumors exploit to evade immune surveillance. The PD-1 receptor on T cells binds to its ligand PD-L1 expressed on tumor cells and immune suppressive cells, leading to T cell exhaustion and reduced cytotoxic activity. In GBM, PD-L1 expression is often upregulated, especially following treatments like radiotherapy, which can induce interferon-γ signaling and subsequent PD-L1 upregulation on tumor cells (151, 152). This immune checkpoint engagement suppresses T cell activation and proliferation, contributing to tumor immune escape. ICIs targeting PD-1 or PD-L1 can restore T cell function, but their efficacy in GBM as monotherapy has been limited due to the highly immunosuppressive tumor microenvironment (153, 154). Moreover, intrinsic resistance mechanisms, such as tumor-intrinsic PD-1 signaling promoting glioma growth independently of PD-L1 ligation, further complicate therapeutic outcomes (155). Therefore, combining ICIs with treatments that modulate the tumor microenvironment or enhance immune cell infiltration is critical to improving responses.

The potential synergistic effects of combination therapies have been explored in both preclinical and clinical settings. For instance, the use of oncolytic viruses in conjunction with ICIs has shown promise in preclinical glioma models, where oncolytic virotherapy increases tumor-infiltrating lymphocytes and upregulates immune checkpoints, thereby creating a rationale for combining with checkpoint blockade to enhance antitumor immunity (156). Similarly, nanoparticles combined with low-dose radiation have been shown to recruit macrophages to the tumor site and improve the efficacy of PD-L1 blockade, resulting in improved survival in orthotopic GBM models (157). Clinical trials investigating combinations of ICIs with radiotherapy or chemotherapy have reported mixed results, with some evidence suggesting that neoadjuvant plus adjuvant pembrolizumab may improve overall survival and progression-free survival in recurrent GBM, although the data remain limited and of low certainty (158). Importantly, the timing and sequencing of combination therapies are crucial, as immune-related gene expression after irradiation show time-dependent patterns that may influence the optimal scheduling of ICIs (152). These findings underscore the necessity of well-designed clinical trials that consider the dynamic tumor-immune interactions and incorporate biomarkers to select patients most likely to benefit from combination approaches.

Furthermore, targeting multiple immunosuppressive mechanisms simultaneously may yield additive or synergistic effects. For example, combination of ICIs with agents targeting myeloid-derived suppressor cells and combination of ICIs with glucose transporter 1 (GLUT1) inhibition have both been proposed to alleviate the immunosuppressive tumor microenvironment and enhance T cell-mediated tumor clearance (159, 160). Additionally, epigenetic modulators like histone deacetylase (HDAC) inhibitors have been identified as enhancers of macrophage phagocytosis and may potentiate the efficacy of checkpoint blockade in GBM (161). These combinatorial strategies aim to shift the balance from immune tolerance to immune activation within the tumor milieu.

### 6.3 Synergistic effects of other therapies

The integration of radiotherapy, chemotherapy, and interstitial immunotherapy represents a promising combinatorial strategy to overcome the therapeutic resistance of glioblastoma. Radiotherapy (RT) and chemotherapy, primarily temozolomide (TMZ), remain the standard of care following maximal surgical resection; however, their efficacy is limited by tumor heterogeneity and an immunosuppressive tumor microenvironment (162, 163). Radiotherapy not only induces DNA damage to eliminate tumor cells but also modulates the immune response, thereby potentially augmenting the effects of immunotherapy (163). Notably, the choice of radiation regimen impacts immune activation; for example, single-dose radiotherapy (10 Gy) elicits greater lymphocyte infiltration and synergizes more effectively with anti-PD-1 therapy compared to fractionated doses, suggesting that optimized radiation schedules can potentiate immunotherapeutic responses in GBM (164). Chemotherapy with cyclophosphamide exerts immunomodulatory effects, including depletion of regulatory T cells, which may enhance the efficacy of immune checkpoint blockade (ICB) when administered in combination (165). Neoadjuvant immune checkpoint inhibitor therapy has been shown to diversify the T cell receptor repertoire and increase tumor-infiltrating lymphocyte clonotypes (166). These findings underscore the rationale for combining RT, chemotherapy, and immunotherapy in a temporally optimized manner to maximize immunogenic tumor cell death and immune system activation.

Radiotherapy can induce immunogenic cell death, increasing the release of tumor-associated antigens and danger-associated molecular patterns that recruit and activate dendritic cells and T lymphocytes. Concurrently, combination therapy with dendritic cell vaccines and anti-PD-1 has been reported to induce robust CD4+ and CD8+ T cell responses in GBM patients (165). The combined approach may modulate tumor-associated macrophages to promote their polarization toward a pro-inflammatory M1 phenotype; in addition, targeting ARPC1B combined with immune checkpoint blockade can reverse pro-tumor macrophage polarization and reshape the immunosuppressive microenvironment (167, 168). For instance, cathepsin B-responsive delivery systems have been developed to simultaneously target glioma cells and repolarize macrophages from the M2 immunosuppressive phenotype to the M1 proinflammatory phenotype, enhancing chemo-immunotherapy efficacy (167). These multimodal therapies have demonstrated synergistic antitumor effects in preclinical models, highlighting the importance of integrating various treatment modalities to overcome the immunosuppressive barriers in GBM.

Evaluating the efficacy and managing the toxicities of combination therapies remain critical for clinical translation. Clinical trials combining immunotherapy with RT and chemotherapy have reported immune modulation within the tumor microenvironment, such as increased lymphocyte infiltration and diversified TCR repertoires, although overall survival benefits have been modest thus far (5, 162). The addition of agents like bevacizumab to RT or chemotherapy has shown potential in reducing radiation-induced adverse effects such as radiation necrosis and pseudoprogression, thereby improving tolerability (169). Moreover, combination regimens involving dendritic cell vaccines with anti-PD-1 antibodies and immunostimulatory agents like poly I:C have been well tolerated and elicited robust antitumor T cell responses without significant immunotherapy-related adverse events (165). However, careful monitoring and management of immune-related adverse events remain imperative, especially when combining multiple immunomodulatory agents. The development of imaging biomarkers, such as dynamic granzyme B PET, may provide non-invasive tools to assess immune activation and therapeutic response, facilitating personalized treatment adjustments (170). Ultimately, optimizing dose schedules, sequencing, and combination partners is essential to maximize therapeutic efficacy while minimizing toxicity in GBM patients undergoing multimodal treatment.

### 6.4 Major challenges in clinical translation

The clinical translation of meningeal immunity-based interstitial therapy for glioblastoma faces several formidable challenges, spanning technical, safety, and clinical trial design aspects. At the technical level, precise modulation of the meningeal immune system and efficient delivery of immune cells to the central nervous system remain critical hurdles. The meningeal lymphatic vessels have recently been recognized as pivotal conduits for CNS immune surveillance, antigen drainage, and initiation of anti-tumor immunity (113, 117). However, the complexity of the meningeal immune environment requires sophisticated strategies to selectively enhance beneficial immune responses without triggering deleterious inflammation. For instance, emerging nanotechnologies have been engineered to exploit MLVs for targeted drug delivery and immune modulation, such as metal-supramolecular assemblies that bypass the blood-brain barrier via MLVs to convert immunologically “cold” GBM tumors into “hot” ones by reprogramming tumor-associated macrophages and enhancing dendritic cell trafficking (114). Despite these advances, ensuring precise spatiotemporal control over immune activation in the meninges, while minimizing systemic exposure, remains a significant technical challenge. Moreover, the delivery of immune effector cells such as CAR-T cells to intracranial tumor sites is impeded by the BBB and the immunosuppressive tumor microenvironment (171). Optimizing delivery routes, cell engineering, and homing mechanisms is essential to overcome these barriers and achieve effective therapeutic concentrations within the CNS.

In addition to technical barriers, biological safety and management of immune-related adverse events constitute major concerns in clinical translation. Immunotherapies such as CAR-T cell therapy targeting GBM can cause non-critical neurological adverse events (172). For example, CAR T-cell therapies have demonstrated relative safety but marginal efficacy in GBM patients, with a notable incidence of cytokine release syndrome and neurological toxicities (172). The meningeal immune system’s dual role in maintaining CNS homeostasis and mediating immune responses necessitates careful modulation to avoid disrupting physiological functions. Furthermore, the immunosuppressive milieu within GBM contains both disease-specific suppressive granulocytes and dysfunctional dendritic cells, both of which complicate the induction of robust anti-tumor immunity (173, 174). Therefore, risk mitigation strategies, including precise dosing, monitoring biomarkers of neurotoxicity, and employing controllable or localized immunomodulatory approaches, are crucial for clinical safety.

Clinical trial design for meningeal immunity-based therapies in GBM also presents unique challenges, particularly in patient selection, efficacy evaluation, and endpoint standardization. GBM has substantial inter- and intra-tumoral heterogeneity in immune microenvironment composition (175). Moreover, the establishment of standardized criteria for assessing therapeutic efficacy is complicated by the difficulty in distinguishing tumor progression from treatment-induced inflammation or pseudoprogression on imaging studies. Immune cell infiltration scoring systems and advanced imaging modalities, such as dynamic contrast-enhanced MRI and PET tracers for glymphatic function, are being developed to provide more accurate and reproducible evaluation metrics (124, 175). Local regional nanotechnology-based strategies targeting the glioblastoma resection margin have been proposed as a potential approach to improve treatment outcomes. The recruitment of patients with adequate performance status and the management of confounding factors, such as corticosteroid use and prior therapies, further complicate trial execution. These challenges underscore the need for multidisciplinary collaboration and innovative trial designs, including window-of-opportunity studies and adaptive protocols, to accelerate clinical translation.

**Figure 5:**
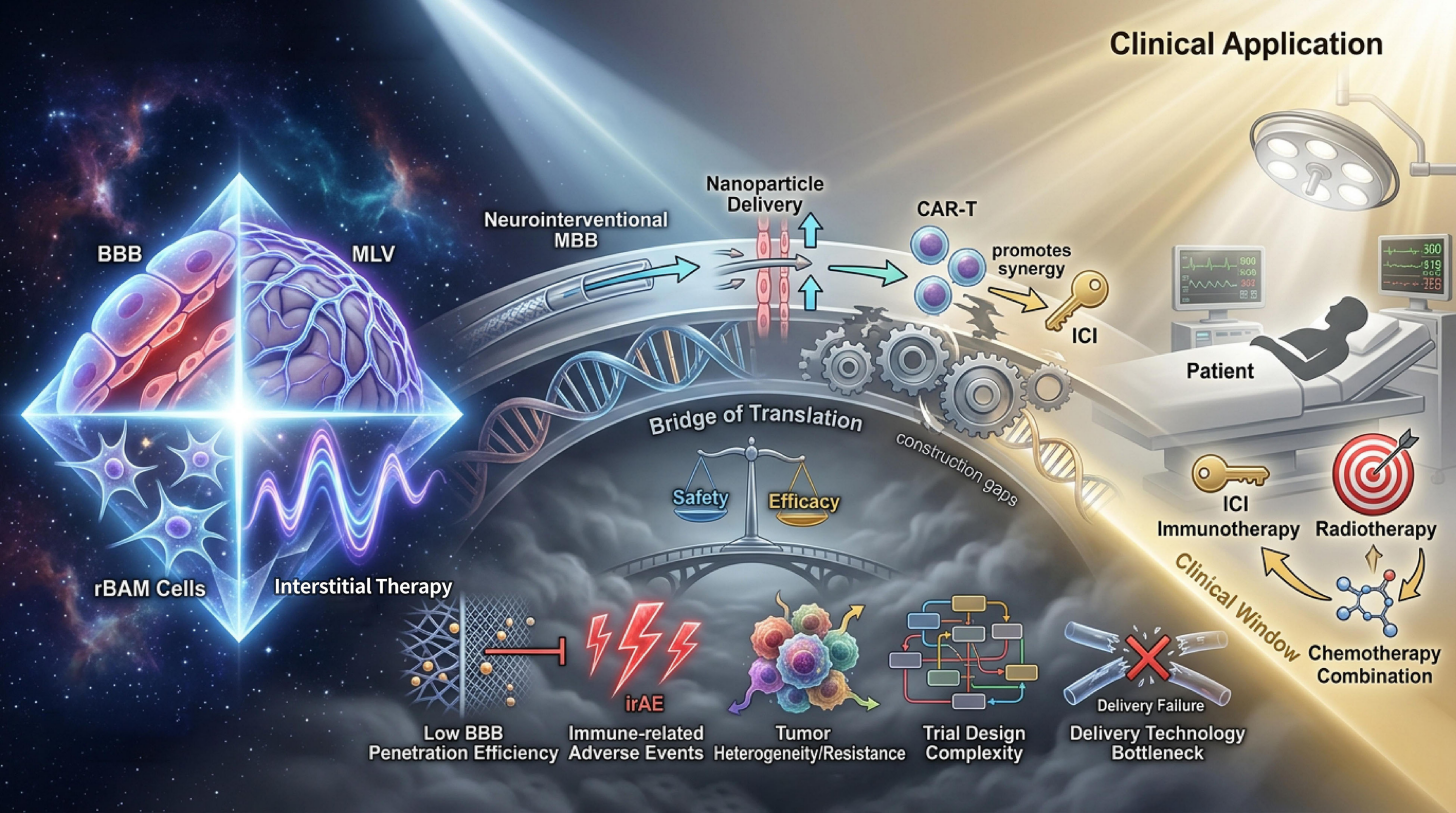
Clinical translation of “Interstitial” immunotherapy: strategies, bottlenecks, and prospects.

Meningeal vascular embolism: The technology is mature and safety is controllable in conventional cerebrovascular disease applications, but there is no direct clinical evidence for its use in GBM immune regulation, and it remains at an exploratory application stage. ICIs combination therapy: Monotherapy is ineffective for GBM; clinical results of combination with chemoradiotherapy are inconsistent; only neoadjuvant + adjuvant Pembrolizumab may improve survival in recurrent GBM, with low evidence level and small sample size. Multimodal combination therapy: Radiotherapy + chemotherapy + immunotherapy combination is well-tolerated and can improve the tumor immune microenvironment, but overall survival benefit is limited; lacks large-scale clinical trial data and is overall at a clinical exploratory stage. Collectively, these challenges highlight the complexity of translating meningeal immune system modulation into effective clinical therapies for GBM. Addressing the technical difficulties of precise immune modulation and cell delivery, ensuring biological safety amidst the CNS’s unique environment, and developing robust clinical trial frameworks are critical steps toward realizing the potential of this novel therapeutic paradigm. Continued advances in nanotechnology, immune engineering, and imaging, coupled with deeper understanding of meningeal immunobiology, are expected to progressively overcome these barriers and enable successful clinical application (**Figure 5**).

## 7 Conclusion

The exploration of meningeal immunity and its therapeutic implications for glioblastoma represents a paradigm shift in neuro-oncology, moving beyond the confines of the central nervous system as an immunologically privileged site. This review has synthesized evidence highlighting the formidable barriers—the blood-brain barrier, the profoundly immunosuppressive tumor microenvironment, and the paucity of effective immune cell infiltration—that have historically rendered GBM a graveyard for conventional immunotherapies. The discovery of the meningeal lymphatic system and resident border-associated macrophages has not only redrawn the immunological map of the CNS but has also provided a compelling mechanistic foundation for novel intervention strategies. Among these, “Interstitial” Therapy, which strategically targets the tumor-meningeal interface, emerges as a particularly innovative approach designed to leverage the unique properties of the meningeal immune compartment to potentiate anti-tumor responses.

From an expert perspective, the development of this field underscores a critical evolution in our understanding: effective CNS immunity requires a dual focus on both the intratumoral space and its peripheral immunological gateways. The meninges are no longer seen as mere protective coverings but as active immunological hubs that can be harnessed. The “Interstitial” Therapy strategy intelligently sidesteps some of the most intractable problems of direct intratumoral delivery. By targeting the peritumoral region and leptomeningeal spaces, it potentially facilitates more efficient immune cell trafficking, antigen presentation via meningeal lymphatic vessels, and modulation of the local immune milieu by rBAMs and other meningeal-resident cells. This approach reframes the challenge from one of brute-force penetration to one of strategic engagement at a more accessible and immunologically active border.

However, the translation of this promising concept into clinical reality necessitates a balanced and critical analysis of competing research perspectives and findings. A primary area for balance is between the enthusiasm for new targets and a sober assessment of safety. The induction of controlled inflammation in the meninges to stimulate immunity carries inherent risks, such as meningeal vascular complications (e.g., thrombosis or embolism) and neuroinflammatory side effects. Research must therefore proceed in parallel: optimizing therapeutic efficacy while rigorously defining the safety window through advanced imaging and biomarker studies. Furthermore, the role of rBAMs appears complex and potentially context-dependent, with studies suggesting both pro- and anti-tumor functions. A nuanced understanding is required to determine whether these cells should be recruited, reprogrammed, or depleted in different therapeutic contexts.

Another crucial balance lies in integrating “Interstitial” Therapy strategies with the broader arsenal of oncology treatments. It is unlikely to be a standalone cure. Its greatest impact will likely be realized in rational combination with other modalities. For instance, combining meningeal-focused therapies with systemic immune checkpoint inhibitors could create a synergistic effect: the former may promote T-cell priming and trafficking to the CNS border, while the latter could reverse exhaustion within the TME. Similarly, its integration with standard-of-care treatments like tumor-treating fields (TTFields) or targeted radiotherapies needs exploration, as these may alter BBB permeability and the immune landscape, potentially enhancing the efficacy of “Interstitial” Therapy interventions. The technical challenges of reliable delivery to the leptomeningeal space—via intrathecal routes, convection-enhanced delivery, or novel biomaterial systems—also require significant interdisciplinary innovation.

In conclusion, the study of meningeal immunity has irrevocably changed the landscape of GBM immunotherapy, offering a fresh set of targets and a new philosophical approach centered on the tumor’s immunological periphery. The “Interstitial” Therapy strategy embodies this new thinking, holding substantial promise for overcoming historical barriers. Future progress hinges on a balanced research agenda that meticulously addresses safety concerns, unravels the dualistic nature of meningeal immune cells, and pursues intelligent combination therapies. As our knowledge of this intricate interface deepens, the potential extends beyond GBM, paving new paths for immunotherapy against a range of CNS malignancies, metastatic brain tumors, and even neuroinflammatory and neurodegenerative diseases. The journey from mechanistic insight to clinical breakthrough is fraught with challenges, but the meningeal gateway now stands open, offering a renewed and strategic avenue for attack against some of medicine’s most formidable neurological foes (**Figure 5**).
